# Oral rehydration therapy training and clinical simulation experience to improve students’ confidence in preparation for international medical mission trip to Nicaragua

**DOI:** 10.1080/21614083.2017.1310419

**Published:** 2017-04-03

**Authors:** Jeremie D. Oliver, John Bachman

**Affiliations:** ^a^ Mayo Clinic School of Medicine, Mayo Clinic College of Medicine, Mayo Clinic, Rochester, MN, USA; ^b^ Division of Family Medicine, Mayo Clinic, Rochester, MN, USA

**Keywords:** Medical educationoral rehydration therapymedical humanitarianism

## Abstract

In order to better prepare the medical, graduate and undergraduate students participating in our international medical mission trip to Nicaragua, we prepared and presented a discussion on Oral Rehydration Therapy (ORT) prior to the international experience. Additionally, a clinical simulation experience was incorporated into our pre-departure training to instruct the students on basic clinical skills, medical interviewing and teamwork. As a tool to measure the students’ prior knowledge on the topic of ORT, we designed a questionnaire, which was distributed and collected prior to the training and simulation experience. Finally, one day after the ORT training and simulation experience, we distributed the same questionnaire and collected the results in order to measure the efficacy of the training and simulation on the students’ perspective of confidence, as well as the students’ retention of the information and skills taught. Our study population consisted of 9 first-year medical students, 1 graduate student, and 16 pre-medical students, all of whom participated in all aspects of the study. In the pre-training survey, only five of the students indicated that they could confidently and effectively explain or administer ORT to someone else. After collecting the results from the post-training survey, all of the students indicated that they could confidently and effectively explain or administer ORT to someone else. We concluded that the ORT training and clinical simulation experience, wherein students could actively apply the knowledge they gained on the administration of ORT to patients, are effective tools to aid in the preparation of medical, graduate and undergraduate students by way of increasing students’ level of confidence in the subject material prior to embarking on international medical mission trips.

## Introduction

Many pre-medical, medical and graduate students in the biomedical sciences share great enthusiasm for participating in international medical humanitarian work prior to beginning any significant formal medical training, particularly in direct patient care for specific critical conditions, such as severe dehydration [1]. We have conducted a pilot study to evaluate the efficacy of clinical didactic training and simulation instruction in Oral Rehydration Therapy (ORT) to increase students’ level of confidence to perform tasks in preparation for an international medical mission trip to Nicaragua. We prepared and presented a discussion on ORT before the international experience. Additionally, a clinical simulation experience was incorporated into our pre-departure training to instruct the students on basic clinical skills, medical interviewing and teamwork. Our main objectives in conducting this study were to prepare pre-medical, medical and graduate students for direct patient interaction in the mission trip to Nicaragua and to define potential areas of improvement for future didactic and simulation training of medical humanitarian workers, as well as to assess students’ confidence in their ability to perform clinical tasks in the context of ORT, following an interactive didactic training that included a simulation exercise.

## Methodology

We designed a clinical and didactic training session for all study participants to receive as a part of the preparation for the humanitarian healthcare mission trip to Nicaragua. The three-hour training session was divided into three parts: a hands-on didactic portion, detailing the clinical skills and knowledge necessary for the administration of ORT; a simulation exercise, wherein hands-on clinical skills in small group teams were taught to complement the didactic knowledge of specific indications for ORT administration; and a debriefing session that consisted mainly of a recap of the concepts that had been taught in the didactic session and clinical simulation. We tried to answer any lingering questions the students had and to help connect the knowledge they had been given on the science behind the supplements commonly used in ORT practice and the clinical signs and symptoms of moderate to severe dehydration. They had to understand how to administer ORT in a low-resource setting. This format of training session has been utilised in past preparations for medical mission trips from our institution, and has been shown to be successful in promoting participants’ confidence in working as part of a medical team, delegating tasks and being a leader [2].

As a tool to measure the students’ prior knowledge on the topic of ORT, we designed a questionnaire, which was distributed and collected before the training and simulation experience. Finally, one day after the ORT training and simulation experience, we distributed the same questionnaire and collected the results in order to measure the efficacy of the training and simulation, as well as the students’ retention of the information and skills taught. Our study population consisted of 9 first-year medical students, 1 graduate student, and 16 pre-medical students, all of whom participated in all aspects of the study. Twenty six participants completed a questionnaire before the training sessions, and 18 of the participants completed the same questionnaire after taking part in the training sessions (see Tables 1 and 2). The medical trip to a small town near Esteli, Nicaragua, was organised through Global Brigades (www.globalbrigades.org). Each student-doctor team had an interpreter who was either a student with several years of Spanish-speaking experience or a local Nicaraguan with significant English-speaking ability. More than 1000 patients were seen and treated over four days.

**Table 1.** Pre-training questionnaire.

Questions	“Yes” answers	“No” answers	“N/A” answers
Have you ever heard of Oral Rehydration Therapy (ORT) before?	15	11	–
Do you know what ORT is used for?	15	11	–
Have you ever used ORT before?	5	21	–
From what you currently know about ORT, do you find it to be useful?	11	–	15
Do you feel you could recognise the signs/symptoms of dehydration?	22	4	–
Do you know how to prepare a homemade solution for ORT?	6	20	–
Can you administer ORT without potable water?	9	17	–
Do you know what should be done if a patient vomits whilst administering ORT?	2	24	–
Should supplemental nutrition be given whilst on ORT?	16	10	–
Do you feel like you could effectively explain or administer ORT to someone else?	5	21	–

**Table 2.** Post-training questionnaire.

Questions	“Yes” answers	“No” answers	“N/A” answers
Have you ever heard of Oral Rehydration Therapy (ORT) before?	18	0	–
Do you know what ORT is used for?	18	0	–
Have you ever used ORT before?	11	7	–
From what you currently know about ORT, do you find it to be useful	18	0	–
Do you feel you could recognise the signs/symptoms of dehydration?	17	1	–
Do you know how to prepare a homemade solution for ORT?	18	0	–
Can you administer ORT without potable water?	17	1	–
Do you know what should be done if a patient vomits whilst administering ORT?	18	0	–
Should supplemental nutrition be given whilst on ORT?	16	2	–
Do you feel like you could effectively explain or administer ORT to someone else?	18	0	–

## Results

**Figure 1.**
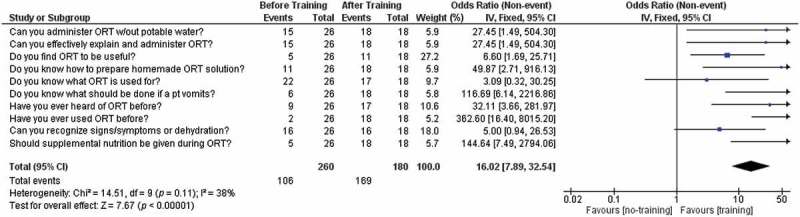
Forest plot of questionnaire results, meta-analysis.

In the pre-training survey, only five of the students indicated that they could confidently and effectively explain or administer ORT to someone else. After collecting the results from the post-training survey, all of the students indicated that they could confidently and effectively explain or administer ORT to someone else. Utilising Review Manager 5.3, we conducted a meta-analysis of the pooled results of the pre-training and post-training questionnaires. Upon statistical analysis of the questionnaire results before and after the didactic and clinical training in ORT, there was a correlation among the students who have been formally trained in ORT to assess their knowledge of clinically-relevant ORT practice with greater confidence, as defined by the questionnaire (Mean difference: 16.02; 95%CI 7.89–32.54; *p* < 0.00001) (see Figure 1). Increased self-scored competency in ORT knowledge and clinical application is seen in meta-analysis of those students who completed the questionnaire after being fully-trained.

## Discussion

The purpose of this study was to assess the impact on students’ confidence in their ability to perform clinical tasks in the context of ORT following an interactive didactic training that included a simulation exercise. It was designed to prepare them for an international medical mission trip. Participants reported increased confidence in their ability to administer and teach someone how to administer ORT, prepare ORT solution, recognise the signs and symptoms of moderate to severe dehydration and effectively treat patients with ORT technique. Several studies have found similar results to our study, in the context of evaluating students’ perspectives in their training, including a study conducted by Kameg et al. in 2013, which similarly evaluated the effects of simulation-based training on student retention of knowledge in a clinical setting and performance in hands-on evaluation [3]. While these previous studies did show large improvements in confidence after completing simulation exercises, none was conducted in the context of a medical trip. Moreover, most of these studies evaluated medical students in their third and fourth years of training, thus focusing on a different demographic of student learners [4]. The results of our study support the use of clinical simulation experiences in pre-medical students, first-year medical students and biomedical graduate student education.

Our study had several limitations. It was small, non-randomised and involved only one institution. Therefore, the findings may not be able to be generalised. Additionally, our study followed participants only until the end of the simulation and training experience. We would suggest follow-up over the academic year with regards to the students’ confidence in their didactic and clinical knowledge of ORT, basic clinical skills, medical interviewing and teamwork. Further studies on the objective efficacy of clinical simulation, measured on the basis of more objective assessment of students’ prior knowledge before the clinical simulation and didactic learning exercises and concluding knowledge following the training experience, would be warranted to directly assess the impact of such clinical and didactic training on students’ knowledge of the subject material before an international medical mission trip. This study focused on the assessment of the students’ self-scored confidence levels before and immediately following clinical simulation and didactic training. The medical mission trip to Nicaragua provided many challenges, including having to contend with limited resources and a large number of patients, which we were not able to replicate in our simulation exercise and which effectively gave participants the opportunity to work together in stressful situations and handle demanding schedules. Further studies to evaluate the efficacy of simulation exercises on the outcome of participant stress levels and confidence in handling difficult situations in healthcare would be of great interest.

## Conclusion

Simulation has become an integral and essential part of healthcare training. It has the capability to prepare a wide range of learners for many experiences, as demonstrated by our diverse spectrum of student learners in this study. Although real-life experience may be more beneficial for team building, simulations are an effective means of initiating hands-on training and increasing students’ self-assessed confidence in specific skill sets, particularly in subject areas less familiar to the learners. Our study shows that simulation is effective in improving the learners’ confidence levels preparatory to an international medical trip, in which specific didactic and clinical training in ORT is delivered in the context of medical humanitarianism internationally.
